# Oncogenomic Changes in Pancreatic Cancer and Their Detection in Stool

**DOI:** 10.3390/biom12050652

**Published:** 2022-04-29

**Authors:** Heidelinde Sammallahti, Virinder Kaur Sarhadi, Arto Kokkola, Reza Ghanbari, Sama Rezasoltani, Hamid Asadzadeh Aghdaei, Pauli Puolakkainen, Sakari Knuutila

**Affiliations:** 1 Department of Pathology, Faculty of Medicine, University of Helsinki, 00014 Helsinki, Finland; heidelinde.sammallahti@helsinki.fi; 2 Department of Surgery, Abdominal Center, Helsinki University Hospital and University of Helsinki, 00290 Helsinki, Finland; arto.kokkola@hus.fi (A.K.); pauli.puolakkainen@hus.fi (P.P.); 3 Department of Oral and Maxillofacial Diseases, Helsinki University Hospital and University of Helsinki, 00290 Helsinki, Finland; virinder.sarhadi@helsinki.fi; 4 Digestive Oncology Research Center, Digestive Diseases Research Institute, Tehran University of Medical Sciences, Tehran P.O. Box 1411713135, Iran; r.ghanbari98@gmail.com; 5 Foodborne and Waterborne Diseases Research Center, Research Institute for Gastroenterology and Liver Diseases, Shahid Beheshti University of Medical Sciences, Tehran P.O. Box 1985717411, Iran; samasoltani70@gmail.com; 6 Basic and Molecular Epidemiology of Gastrointestinal Disorders Research Center, Research Institute for Gastroenterology and Liver Diseases, Shahid Beheshti University of Medical Sciences, Tehran P.O. Box 1985717411, Iran; hamid.assadzadeh@gmail.com

**Keywords:** pancreatic cancer, oncogenomics, genomic biomarkers, stool screening, non-invasive screening, early diagnosis, gut microbiota

## Abstract

Pancreatic cancer (PC) is an aggressive malignancy with a dismal prognosis. To improve patient survival, the development of screening methods for early diagnosis is pivotal. Oncogenomic alterations present in tumor tissue are a suitable target for non-invasive screening efforts, as they can be detected in tumor-derived cells, cell-free nucleic acids, and extracellular vesicles, which are present in several body fluids. Since stool is an easily accessible source, which enables convenient and cost-effective sampling, it could be utilized for the screening of these traces. Herein, we explore the various oncogenomic changes that have been detected in PC tissue, such as chromosomal aberrations, mutations in driver genes, epigenetic alterations, and differentially expressed non-coding RNA. In addition, we briefly look into the role of altered gut microbiota in PC and their possible associations with oncogenomic changes. We also review the findings of genomic alterations in stool of PC patients, and the potentials and challenges of their future use for the development of stool screening tools, including the possible combination of genomic and microbiota markers.

## 1. Introduction

Pancreatic cancer (PC) is known for its aggressive course, having the highest mortality of all major cancers, and a 5-year survival rate of only around 10% [1,2]. PC was the fourth most common cause of cancer-related deaths in the US in 2019 [1], and has been projected to advance to the second leading cause by the year 2040 [3]. On a worldwide level, PC mortality is expected to almost double during the next 40 years [4]. Established risk factors for PC are, amongst others, obesity, tobacco and alcohol consumption, diabetes mellitus, chronic pancreatitis (CP), and hereditary factors [5].

An enormous challenge in the management of PC is the late appearance and the non-specificity of symptoms [6]. Consequently, the disease is frequently detected at a locally advanced or metastasized stage, which make the tumors unsuitable for surgical resection [7]. Due to the relatively low incidence of this malignancy, screening of the general population is not considered applicable [8]. To date, there is no single diagnostic test to definitively identify PC. In fact, a series of different images and biopsies are needed, and these procedures are usually performed only after the onset of symptoms. Unfortunately, no approved tests for the early detection of PC are currently available [9].

Since surgery combined with effective adjuvant therapy represents the only potential curative treatment option for patients with early-stage pancreatic adenocarcinoma [10], the development of early screening methods is urgently needed for the improvement of survival rates.

In the past few years, research on PC diagnostics has been focusing on the discovery and evaluation of novel molecular biomarkers. These include serum markers such as enzymes, cytokines, antibodies and antigens, and moreover, nucleic acid-based markers typical for PC [11,12,13]. Oncogenomic alterations, such as gene mutations, epigenetic, and transcriptomic changes present in tumor tissue, can also be detected from body fluids in circulating tumor cells (CTCs), circulating tumor DNA (ctDNA), or cell-free DNA and RNA (cfDNA and cfRNA) [14]. Although it is often not clear whether the alterations in nucleic acids detected in body fluids are of free or cellular origin, these nucleic acids have been considered to largely originate from tumor tissue and may therefore be utilized as biomarkers for early screening, prognosis, disease monitoring, and prediction of treatment outcome in PC through the non- or semi-invasive analysis of different body fluids, such as saliva, pleural fluid, urine, sputum, stool, or plasma. When referring to genomic alterations detected in body fluids in this review, these alterations may be cell-free and/or cellular nucleic acids in origin.

Another approach to finding new biomarkers is the analysis of the microbiota. In PC, distinct microbial profiles have been found in various locations and body fluids, including pancreatic tumor tissue, duodenal mucosa, saliva, pancreatic fluid, pancreatic cyst fluid, and stool [15]. Altered microbiota have been brought into connection with oncogenomic changes and PC tumorigenesis [16]. Certain microbes or their metabolites can induce cancer mutations or have effects on epigenetics and micro-RNA (miRNA) expression, and reciprocally, host genetics and cancer mutations may have an impact on microbiota composition and diversity, which we reviewed recently [17]. Distinct, PC-associated microbial profiles could, therefore, be used as biomarkers for PC [12,18,19]. By stool analysis of PC patients, both the alterations in gut microbiota as well as oncogenomic alterations could be detected and their associations could be studied, which could lead to the detection of novel biomarkers for the non-invasive screening of PC.

In the present review, we explore the oncogenomic features that have been identified in tumor tissue as well as in stool DNA of patients with PC. In addition, the relationship between PC driver mutations and microbiota shall be discussed. Furthermore, we investigate the potential of oncogenomic and microbial alterations in stool as PC biomarkers in clinical research and clinical practice. For this, literature was searched from NCBI’s PubMed [20], using the key words “genomic”, “pancreatic cancer”, “stool” and “fecal”, and relevant original articles were selected from these and from cross-references.

## 2. Alterations in Tumor Tissue

### 2.1. Chromosomal Aberrations

#### 2.1.1. Cytogenetics

Pancreatic ductal adenocarcinoma (PDAC), which accounts for more than 90% of all PC cases [21], has a highly complex cytogenetic profile that involves all chromosomes and includes both numerical and unbalanced structural aberrations [22,23]. This high complexity and the extensive intratumor cytogenetic heterogeneity are assumed to be a consequence of the advanced disease stage at the point of cytogenetic analysis [23]. In an analysis of six cytogenetic studies including 127 PDAC cases, several recurrent numerical aberrations were observed, including monosomy 18 (in 60% of cases), monosomies 4, 6, 9, 12, 17, 21, 22, X and Y, and trisomies 7 and 20 (in 25–38% of cases). The most common recurrent breakpoints were 13q10, 19q13, 1q10, 8q10, 14q10, 17p11 and 17q10, with frequencies of up to 13% [23]. A more recent cytogenetic study that included 48 PDAC cases detected deletions on 17p, 18q, 21q, and the pericentromeric region of chromosome 18 (CEP18), and gains on 7q and 20q [24].

#### 2.1.2. Array-Comparative Genomic Hybridization (aCGH)

Corbo et al. summarized eleven aCGH studies, including 249 PDAC cases, in which high frequency gains were observed on chromosome arms 8q, 20q, 17q, 7p, 7q and 5p, and high-frequency losses on 9p, 18q, 8p, 17p and 4q. In twelve CGH and array CGH studies comprising 320 cases of PDAC cell lines and cancer tissues, variable gains and losses across the whole genome were seen, with the highest frequencies of gains on 5p, 7p and q, 8p and q, 11q, 12p, 19q and 20q, and of losses on 1p, 3p, 4q, 8p, 9p, 13q, 17p, 18q and 21q. In addition, frequent fold-back inversions in metastases were reported, which were suggested to be caused by breakage–fusion–bridge cycles after telomere loss [22]. Birnbaum and colleagues analyzed 39 PDAC cases by high-resolution array CGH and found frequent gains on 1q, 3q, 5p, 6p, 7q, 8q, 12q, 15q, 18q, 19q and 20q, and losses on 1p, 3p, 4p, 6, 8p, 9, 11q, 15q, 17, 18, 19p, 20p, 21 and 22 [25]. A large-scale study by the Cancer Genome (TCGA) Research Network that included 150 PDAC patients detected arm-level somatic copy number aberrations (SCNAs) in one third of cases. Of these, amplification of 1q was detected in 33%, deletions of 6p and 6q in 41% and 51%, of 8p and 9p in 28% and 48%, of 17p and 17q in 64% and 31%, and of 18p and 18q in 32% and 71% of cases, respectively [26]. 

The International Cancer Genome Consortium (ICGC) and the Australian Pancreatic Cancer Genome Initiative (APGI) analyzed 100 PDAC cases by whole-genome sequencing (WGS) and copy number variation (CNV) analysis [27]. Close to 12,000 somatic structural variants were detected, the majority (85.2%) of which were intrachromosomal, including inversions, deletions, fold-back inversions, amplified inversions, tandem duplications, duplications, and other intrachromosomal rearrangements. Of the structural variants, 14.8% were interchromosomal translocations. More than half (about 6900) of all structural aberrations directly disrupted gene sequences. Recurrent breakpoints were detected in 1220 genes. Overall, 1236 structural variants caused non-recurrent gene fusions, 183 of which were expressed. Based on these findings, PDAC was subtyped into the types stable, scattered, unstable, and locally rearranged [27]. Table 1 summarizes the cytogenetic data of the cited studies.

### 2.2. Gene Mutations

The complexity of PDAC cytogenetics is also reflected in the mutational landscape of this malignancy. A variety of oncogenes, tumor suppressors including DNA damage repair genes, axon guidance, and chromatin modification genes are altered during PDAC tumorigenesis [26,27]. The most prominent genomic feature of PDAC is alterations of the oncogene *KRAS* on chromosome 12p, which are present in more than 90% of cases. In addition, three other genes play a major role in sporadic forms of PDAC, namely the tumor suppressors *CDKN2A*, *TP53* and *SMAD4* on chromosomes 9p, 17p and 18q, respectively. The above-mentioned large-scale integrated genomic characterization of 150 PDAC cases by TCGA Research Network found *KRAS* to be mutated in 93%, *TP53* in 72%, *SMAD4* in 32% and *CDKN2A* in 30% of PC tumor tissue samples [26]. The most common *KRAS* mutations were G12D, G12V and G12R, with frequencies of 41%, 27% and 19%, respectively. In cases with wild-type *KRAS*, mutations in alternative driver genes or in other RAS pathway genes were found. Other mutations with incidences between 1% and 9% were detected in the tumor suppressor *PTEN*, the oncogenes *GATA6*, *GNAS*, *AKT2*, *FGFR1*, *MYC*, *BRAF* and *MDM2*, the chromatin modification genes *ARID1A*, *PBRM1*, *MLL3* and *MLL4*, and DNA damage-repair genes *BRCA1*, *BRCA2*, *ATM and PALB2* [26]. Waddell et al. showed that disruption of key driver genes and pathways in PDAC are often caused by structural variation [27]. The most commonly amplified or deleted driver genes in PDAC and their chromosomal locations are summarized in Table 1.

Recent progress in genomic research of early-stage PC and its pre-cancerous lesions has resulted in a clearer picture of the progression of mutational changes during PC development. According to these, mutations in *KRAS* are one of the earliest oncogenic alterations, which are already detectable in low-grade pancreatic lesions, whereas mutations in the other major PC driver genes take place only in advanced lesions [29,30]. Such findings should be utilized for the development of screening methods for early PC, and for the differentiation between high-grade and low-grade pancreatic lesions.

Since this review is focusing on oncogenomic findings in tissue and stool and their clinical significance in the early diagnosis of PC, and to our knowledge transcriptomic analysis has not been done from stool (except of miRNA), gene expression profiling of PC tissue or the functional roles of PC driver genes are not going to be discussed here. However, these topics have recently been reviewed elsewhere [31,32,33].

### 2.3. Epigenetic Alterations

In addition to genomic changes, epigenetic alterations play a major part in PC tumorigenesis [34,35]. These occur in the form of methylations, histone modifications, and RNA interference [36]. Representing the most important and most studied epigenetic mechanism, DNA methylation regulates developmental and differential processes in health and disease [35,37]. By the action of DNA methyltransferases (DNMTs), CpG islands in the promotor regions of genes are methylated, leading to transcriptional silencing of the corresponding genes. In cancer, tumor suppressor genes are often hypermethylated and thus silenced by the aberrant function of methyltransferases [38]. Conversely, oncogenes can be activated by hypomethylation through demethylases, such as TET enzymes, leading to increased expression and promotion of tumorigenesis [39,40]. Profiling of ectopic methylations in cancerous tissues enables the detection of novel cancer genes, the prediction of treatment outcome and patient survival, and the development of new diagnostic, prognostic and therapeutic biomarkers [22].

In PC, altered DNA methylations were found to have an impact on gene expression, genome structure reorganization, tumor grade and stage, and patient survival [41]. In the previously mentioned integrated genomic analysis by TCGA Research Network, DNA methylation profiling of 150 PDAC tumor tissue cases in combination with gene expression analysis detected 98 genes that were silenced through hypermethylation, including the supposed tumor suppressors *ZPF82*, *PARP6*, and *DNAJC15* at higher prevalence, and the important cancer genes *BRCA1* and *MGMT* at low prevalence, amongst others [26]. Likewise using PDAC data provided by TCGA, Mishra and colleagues conducted an analysis that integrated global methylation patterns, somatic mutations, CNVs in known oncogenes and tumor suppressors, and gene expression levels [41]. They observed differential methylation in epigenetic regulatory genes, including writer genes such as histone methylation, histone acetylation, and arginine methylation writers. Epigenetic reader genes such as DNA methylation and histone methylation readers were differentially methylated, as well as epigenetic eraser genes such as histone acetylation erasers. In addition, differential DNA methylation was found in genes coding for chromatin remodeling proteins, *ARID1B*, *SMARCA2* and *SMARCD3*, and several histone protein genes. Furthermore, developmental genes, such as homeobox-containing genes of the HOX and PAX families and of the PRRX, MSX and ZEB clusters, were differentially methylated, as well as genes associated with pancreatic development and signaling. Moreover, marker genes that had earlier been associated with patient survival, namely *FOSB*, *KLF6*, *ATP4A* and *GSG1*, were differentially methylated [41,42]. Other methylation studies of PDAC tissue detected overexpression through hypomethylation of the genes *CLDN4*, *LCN2*, *MSLN*, *PSCA*, *S100A4*, *SFN* and *TFF2* [43], transcriptional silencing through hypermethylation of the tumor suppressors *CDKN2A*, *CDKN1C*, *PCDH10*, *RASSF1A*, *CCND2*, *SOCS-1*, and *APC* [44,45,46,47,48], furthermore hypermethylation of *BMP3*, *CNTNAP2*, *EVOLV-4*, *MDF1*, *miR-9-1*, *PENK*, and *ZNF415* [49,50,51], amongst others. By pathway enrichment analysis of differentially methylated genes, enrichment was seen in members of the WNT pathway [48], signaling pathways connected to apoptosis, cell cycle and cell differentiation, cytoskeleton structure, immune- and DNA damage-response, as well as major pancreatic signaling pathways including Notch, Hedgehog and TGF-beta-related genes [41]. An overview of differential methylations in PDAC is shown in Table 2.

### 2.4. Alterations in Non-Coding DNA

Over the past two decades, it has become increasingly apparent that in addition to mutations and epigenetic mechanisms interfering with driver genes, alterations in non-coding DNA (ncDNA) are also an important factor in cancer development, progression, and drug resistance [53]. NcDNA is transcribed into various forms of non-coding RNAs (ncRNAs), such as small interfering RNA (siRNA), micro-RNA (miRNA), and long non-coding RNA (lncRNA), many of which play important roles in gene regulation [54]. In cancer, ncDNA can be affected by mutations, copy number alterations and epigenetic mechanisms. These alterations are cell- and tumor-specific and are manifested as altered expression of the respective ncRNA in tumor tissue, which can be detected by transcriptional analysis [55,56]. Table 3 gives an overview of differentially expressed ncRNAs in PC tissue and their biological functions.

#### 2.4.1. miRNAs

The most extensively analyzed forms of ncRNAs in cancer research are miRNAs. These are very short, single-stranded ribonucleic acids, approximately 22 nucleotides in length, which have key functions in the posttranscriptional regulation of gene expression by controlling cancer-relevant biological processes such as cell proliferation, cell-cycle, migration and invasion, stem-cell differentiation, epithelial to mesenchymal transition (EMT), and apoptosis [55,70]. They represent the most abundant form of ncRNAs, with numbers of human mature miRNAs estimated up to 2300, and are presumed to regulate the expression of around 60% of human genes [71,72,73]. MiRNAs prevent translation or induce mRNA degradation through base-pairing with the 3′-untranslated region (3′-UTR) of their target mRNA [74]. They can act in oncogenic or tumor suppressive ways, depending upon the expression of miRNAs targeting tumor suppressors or oncogenes [55,75,76]. MiRNAs are stable in different body fluids and even so in severe conditions like extreme temperatures, extreme pH levels and extended storage. Because of these properties and the possibility to quantify them in very small sample sizes, they have excellent potential to serve as biomarkers for the detection of pathologic conditions, including cancer [77,78].

Through the simultaneous analysis of a high number of miRNAs with Real-Time PCR, microarrays, or direct sequencing methods, distinct miRNA expression profiles in PC tumor tissue and PC cell lines have been reported in numerous studies. Overexpression of miR-17-5p, -21, and -191, as well as decreased expression of miR-218-2 have been observed in PC as well as in other malignancies including colon, stomach and prostate cancer [79]. MiR-21, -155, -221 and -222 were shown overexpressed in both PC and pancreatic intraductal papillary mucinous neoplasms (IPMNs), a pancreatic neoplasm that can develop into PC [21]. A recent review observed the recurrent up-regulation of miR-21, -155, and -221, and down-regulation of miR-34 and miR-145 in several miRNA expression studies of PC cells or tissues [80]. Combinations of some of these miRNAs could serve as biomarkers to differentiate PC lesions from non-cancerous lesions. Furthermore, overexpression of miR-21, -155, -196a-2, -203, -210 and -222 has been associated with poor outcome [81]. MiR-21 is of special interest, since it functions as an oncogene by down-regulating tumor suppressors *PTEN*, *PDCD4*, *TPM1*, and *TIMP3* [22]. MiR-21 has been associated with tumorigenesis, invasion, metastasis [65,66], mesenchymal transition and stemness of PDAC cells, and has recently been suggested as a biomarker for PDAC aggressiveness [67].

#### 2.4.2. lncRNAs

LncRNAs are non-coding molecules longer than 200 nt, which likewise have important regular functions including gene expression, transcriptional regulation, epigenetic gene regulation, and chromatin remodeling, amongst others [82]. Similar to miRNAs, lncRNAs play important roles in cancer, e.g., by regulating proliferation, invasion, metastasis, cell survival, and angiogenesis, through direct or indirect influence on cancer-related signaling pathways [83]. They can activate or inhibit epigenetic-related proteins by binding to them, control the generation of miRNAs, and promote or inhibit gene transcription by base-pairing and by recruiting transcription factors [84]. LncRNAs are encoded within introns or intergenic regions of genes, with sequences either sense or antisense of the respective genes [59]. The number of lncRNA loci in the human genome has recently been estimated around 96,000 [85]. While they are transcribed in low numbers in healthy individuals, they are often overexpressed in cancer or other pathologic conditions [86]. Comparable to miRNAs, lncRNAs can function in oncogenic or tumor suppressive ways and are up- or downregulated in tumor tissue [87]. Because of their important functions in cancer, differentially expressed lncRNAs have been profiled in various malignancies. Since they are also present cell-free in biological fluids, lncRNAs are potential biomarkers for screening, diagnostics, prognostics or disease monitoring [88]. In PC, important differentially expressed lncRNAs are *HOTAIR*, *HOTTIP*, *H19*, *MALAT1*, *PVT1*, *GAS5*, and *MEG3*, amongst others, as reviewed by [59].

#### 2.4.3. Other ncRNAs

Circular RNAs (circRNAs) are covalently closed loop-shaped single stranded molecules of below 100 nt to over 4 kb in size, and are mostly generated from pre-miRNAs by back-splicing or other spliceosomal activities [89]. Like miRNAs and lncRNAs, they have important functions in the posttranscriptional regulation of gene expression, e.g., by competing with mRNAs, interacting with RNA binding proteins, and sponging miRNAs. They too can act in oncogenic or tumor suppressive ways [59,90]. CircRNAs are differentially expressed in PC, more stable due to their circular shape compared to other ncRNAs, and tissue-specific, which makes them ideal biomarkers and potential therapeutic targets [84]. Likewise, PIWI-interacting RNAs (piRNAs) have been detected differentially expressed in PC. They too have important regulatory functions, being involved in the initiation, progression and metastasis of cancer [59,84,91].

## 3. Alterations in Stool

In this section, we review the findings of oncogenomic alterations in stool of PC patients. The rationale behind the screening of stool is the presence of tumor cells in the large intestine. In addition to CTCs that reach the intestine via the blood stream, pancreatic tumor cells end up in the fecal mass after being exfoliated into the pancreatic juice, which is secreted into the small intestine via the pancreatic ducts [92,93]. Tumor-derived DNA/RNA might thus be present in higher concentrations in stool than in blood, and stool DNA/RNA might, therefore, be suitable for the screening of cancer mutations [94].

### 3.1. KRAS and TP53 Mutations

Since mutations of *KRAS* are the most prominent feature in PC oncogenomics, the majority of studies dealing with PC mutations in stool DNA are focusing on this gene. The earliest study we found aimed at testing whether *KRAS* mutations present in tumor cells that were shed into exocrine pancreatic secretions of PDAC patients could also be detected in stool [95]. Both frozen or FFPE (formalin-fixed, paraffin-embedded) tumor tissue as well as stool samples from patients with benign and malignant pancreatic diseases were analyzed by PCR, phage cloning and plaque hybridization assay for *KRAS* codon 12 mutations. Mutant *KRAS* was detected both in tissue and stool samples of patients with benign and malignant pancreatic disease. The detection rate in tissue was 100% in PDAC, 67% in cholangiocarcinoma (CCA) and 65% in CP cases. In stool samples, mutant *KRAS* was detected in 55% of PDAC, 67% of CCA and 33% of CP cases. The authors showed that the mutations found in stool were identical with the ones found in pancreatic tissue, and concluded that the majority of mutations detected in stool originated from pancreatic cancer cells that had exfoliated into the intestinal lumen and had become part of the fecal mass [95].

Two similar case–control studies compared the mutational status of *KRAS* in tumor tissue and stool samples of patients with benign and malignant pancreatic diseases, using mutant-enriched PCR and reversed dot-plot hybridization in microplates [96], and mutant-enriched PCR with allele-specific capture probes [97]. The authors evaluated the diagnostic potential of mutant *KRAS* for detecting PC in stool samples, compared to carbohydrate antigen (CA) 19–9 and carcinoembryonic antigen (CEA). In the first study, [96] the detection rates of mutant *KRAS* in pancreatic tissue were 91% in PDAC, 71% in periampullary carcinoma (PAC) and 67% in CP, and in fecal samples, 40% in PDAC, 33% in CP and 0% in PAC. Finally, it was shown that the diagnostic sensitivity of mutant *KRAS* was comparable to CEA, but much lower than CA 19–9. The authors concluded that mutant *KRAS* could be used for PC screening in combination with other markers, but was not suitable for differentiating between benign and malignant pancreatic diseases [96]. Almost similar conclusions were obtained in the second study, where the detection rate of mutant *KRAS* in stool was even lower (20% in PDAC and, surprisingly, 40% in CP). Despite this low rate, the authors suggested that fecal analysis should still be considered, as it could improve the diagnosis of PC and as a result increase survival rates [97].

Additionally, a similarly designed study by Pezzilli et al. aimed to evaluate the detection of mutant *KRAS* in blood and feces for the differentiation between benign and malignant pancreatic masses. In this PCR amplification screening analysis, no *KRAS* mutations were detected, neither in blood nor in feces. Notably, simple PCR was used here, which has a lower sensitivity than mutant-enriched PCR and could have been the reason for this result. The authors suggested further studies to identify better genetic markers for PC screening in various biological substances [98].

In contrast to the above-mentioned, several other studies presented comparatively high detection rates of driver gene mutations in stool samples. In a large patient-control study involving 201 patients with PC or benign pancreatic disease (BPD) and 60 healthy controls, stool and pancreatic juice were screened for *KRAS* and *TP53* mutations using PCR-restriction fragment length polymorphism (PCR-RFLP) and PCR-single-strand conformation polymorphism (PCR-SSCP) analyses. The detection rates of mutant *KRAS* in pancreatic juice were 87.7% in PC and 23.5% in BPD, and the rates in stool were 88% in PC, 51.1% in BPD and 19.6% in healthy controls (HCs). Mutant *TP53* was detected in pancreatic juice of 47.4% of PC and 12.5% of BPD cases and in stool of 37.1% of PC and 19.1% of CP cases. Due to the higher sensitivity and specificity of mutant *KRAS* in pancreatic juice compared to the findings in stool, the authors proposed that this trace could be used in PC screening in addition to other methods. Likewise, stool could be screened for both *KRAS* and *TP53* mutations, and in combination with serum CA 19–9 could improve the early diagnosis of PC. In addition to the strikingly high detection rates in stool samples of PC patients, mutated *KRAS* was found in one fifth of healthy controls, which had not been reported before [99]. The same research group later examined the stool and serum of 48 PC patients and 85 controls [100]. Mutant *KRAS* was detected in 77.4% of PC cases and in 18.2% of controls, and mutant *TP53* was detected in 25.8% of PC cases and in 4.71% of controls. The controls in this study were patients with benign digestive disorders, which might explain the mutation rates in this group. The sensitivity and specificity of mutated *KRAS* in stool for prediction of PC were 77.4% and 81.2%, and for mutated *TP53* were 25.8% and 95.3%, respectively. Among the tested serum markers, CA 19–9 and CA 242 had the highest diagnostic values, and these values were improved by simultaneous analysis of mutant *KRAS* in stool. No significant differences in the fecal *KRAS* and *TP53* mutation rates between subgroups of PC with different stages or locations were detected, which indicated that these mutations could play a role in early tumorigenesis [100].

In another study, Hwang et al. assessed stool DNA analysis for the diagnose of IPMN and early-stage PC in 20 patients with benign and malignant pancreatic neoplasia and 20 age- and sex-matched healthy controls (HC). Hybrid capture enrichment and assay of the seven possible *KRAS* variants on codons 12 and 13 by quantitative allele-specific real-time target and signal amplification (QuARTS) was used. At a 90% specificity cutoff, the sensitivity of this assay was 62% for detecting PC and 83% for detecting IPMN [101].

A more recent study reported the use of magnetic nanoparticle trace capture probe and PCR for detection of mutant *KRAS* in stool of patients with benign and malignant pancreatic diseases. In line with the above-mentioned studies, the sensitivity and specificity of this novel methodology were compared to serum CA 19–9 for the detection of pancreatic cancer. Mutant *KRAS* was detected in the stool of 81.8% PC patients and of 18.5% patients with benign pancreatic disease, while none was detected in HC. At a sensitivity of 81.8% and a specificity of 81.5%, the diagnostic values of mutant *KRAS* were even slightly higher than those of CA 19–9. The authors suggested simultaneous analysis with both markers to increase the sensitivity to 97.9% for the screening of PC [102].

Investigating the role of *KRAS* in CRC and PC, Haug et al. screened stool samples of 875 unselected older adults for *KRAS* mutations by mutant-enriched PCR and allele-specific hybridization reaction. The overall prevalence of mutated *KRAS* was 8%. Furthermore, they demonstrated a tentative association between mutant *KRAS* and decreased fecal pancreas elastase 1, a marker for exocrine pancreatic insufficiency. However, no associations were found between mutation state and colonoscopic findings. Based on these results, the authors did not recommend this assay for CRC screening, but suggested considering it for early PC screening together with other markers [103].

### 3.2. Methylations

A different approach was adopted by Kisiel et al. [92], who aimed at finding and evaluating epigenetic markers for the non-invasive screening of PC in stool. Cancer tissue of 24 patients and pancreatic tissue of 30 HC was assayed for the DNA methylation status of nine target genes, *BMP3*, *NDRG4*, *EYA4*, *UCHL1*, *MDFI*, *Vimentin*, *CNTNAP2*, *SFRP2*, and *TFPI2*, by real-time methylation-specific PCR (MSP) in bisulfite-treated DNA. The top four differentially methylated genes, *BMP3*, *EYA4*, *MDFI* and *UCHL1*, with the highest areas under the receiver operating characteristics curves (ROC), were chosen for stool analysis in addition to mutant *KRAS*. Eventually, *BMP3* was the marker that performed best, with significantly higher levels of methylation in stool of pancreatic cancer patients than in HC. *BMP3* acts as a tumor suppressor in colon cancer and has also been found methylated in some types of stomach, breast and lung cancer [104,105]. At a specificity set to 90%, the sensitivity of *BMP3* to detect cases of PDAC was 51%. For the *KRAS* mutations, the sensitivity was 50% at a specificity likewise set to 90%. By combining both methylated *BMP3* and mutated *KRAS*, the resulting Area Under the Curve (AUC) was 0.85, and at 90% specificity, the sensitivity was 67%. No associations were found between tumor stage or site and methylated *BMP3* or mutated *KRAS*. The authors concluded that methylation markers are useful in detecting PC by stool screening, but more investigations are needed to find better combinations of markers, in order to increase sensitivity and specificity, and also to find tools for discriminating between subtypes and different stages of PC [92].

### 3.3. Altered Expression of miRNA

In addition to the mentioned genetic and epigenetic markers, the use of miRNAs as possible biomarkers for PC screening in stool samples has been investigated in a few studies. Link et al. compared the expression levels of miRNA in stool specimens of PC and CP patients with HC. Of a subset of seven miRNAs previously reported to be differently expressed in PC, they found four miRNAs, miR-216a, -196a, -143 and -155, to be under expressed in CP compared to HC, and significantly under expressed in PC compared to HC. The authors demonstrated that miRNAs were highly stable, present at high concentrations and detectable with high reproducibility in stool samples [106]. Similarly, Ren et al. analyzed fecal miRNA expression of 29 PC patients, 22 CP patients and 13 HC. Out of 7 selected miRNAs with differential expression in PC tissue, miR-181b and -210 had higher levels of expression in CP compared to HC. Moreover, miR-181b, -196a and -210 were significantly overexpressed in PC compared to HC. MiR-181b and miR-210 could thus discriminate PC from HC, with sensitivities and specificities of 84.6% and 51.7%, and 84.6% and 65.5%, respectively. In addition, a significant positive correlation between miR-196a in stool and the maximum tumor diameter was observed [107].

In comparable study, Yang et al. investigated the possible use of fecal miRNAs as novel biomarkers for PC [108]. They had a similar approach as the two previously mentioned studies, but also included pancreatic juice, tumor- and normal pancreatic tissue. In summary, five miRNAs with earlier reported differential expression in PDAC tumor tissue, blood, or pancreatic juice, or association with PDAC development (miR-21, -155, -196a, -216 and -217), were analyzed. Out of these, miR-21 and miR-155 had significantly higher, and miR-216 significantly lower levels of expression in primary tumor tissue and pancreatic juice of PC patients, compared to normal adjacent tissue and to pancreatic juice of CP patients. The same expression pattern was observed in the stool samples: miR-21 and miR-155 had significantly higher expressions, whereas miR-216 had lower expressions in stools of PDAC patients compared HC. Diagnostic performance was evaluated by ROC analysis, giving the highest sensitivity, 93.33%, for combined miR-21 and miR-155, with a specificity of 66.67%. The combination of all 3 miRNAs (miR-21, -155 and -216), however, had a better balance between sensitivity and specificity, both at 83.33% [108]. The results of these studies suggest that miRNA expression analysis from stool samples could be an efficient and highly reproducible way of screening for PC, and that the combination of several markers could improve their diagnostic performance. However, validation of selected miRNA biomarkers through large-scale studies would be an important prerequisite for their application [106,107,108]. The findings of pancreatic cancer mutations, methylations and miRNA changes in stool are summarized in Table 4.

### 3.4. Genomic Alterations as Activation of Microbiota Alterations and Vice Versa

It has recently been observed that the microbiome plays an important role in several cancers, including PC [109,110]. Differential microbiota profiles have been detected in the oral cavity, in the pancreas, and in the gut through the analysis of saliva, tumor tissue, pancreatic juice, pancreatic cyst fluid, and stool samples of pancreatic cancer patients [111]. These were, amongst others, increased abundancies of *Porphyromonas gingivalis*, *Graniculatella gingivalis*, *Fusobacterium* [111,112,113] and decreased abundancies of *Neisseria elongata* and *Streptococcus mitis* [113] in the oral cavity, increased abundance of *Fusobacterium* spp., *Malassezia* spp., Firmicutes and Proteobacteria [114,115,116,117,118], and decreased abundance of *Lactobacillus* in the pancreas [114], increased abundance of Gammaproteobacteria [119], *Helicobacter pylori*, *Porphyromonas*, *Prevotella*, *Bifidobacterium*, *Synergistetes* [114,120,121] and decreased abundance of beneficial probiotics and butyrate-producing bacteria in the gut [119,122].

Microbiota can drive PC tumorigenesis through several mechanisms, including epigenetic effects, regulation of miRNA expression, induction of inflammation, DNA damage and mutations [16,123,124], and differential expression of driver genes [125]. Moreover, bacterial metabolites can have an impact on tumorigenesis, e.g., bacterial lipopolysaccharide can initiate carcinogenesis by the hyperstimulation of mutant *KRAS* [126,127]. On the other hand, host genetics [128] and oncogenomic changes such as mutant *KRAS* may influence the diversity and composition of pancreatic and gut microbiota [121]. In the light of these phenomena, which we have discussed in our recent review [17], it would be reasonable to combine the analysis of genomic changes with the analysis of microbiota changes in stool, in order to find out more about the interconnections between cancer mutations and microbiota alterations. To our knowledge, this has not been done in pancreatic cancer.

## 4. Clinical Significance of Genomic Alterations in Stool

Besides more effective treatment regimes, a crucial factor for the improvement of PC outcome would be the development of strategies for the diagnosis of early-stage PC, or even precancerous lesions prior to malignant transformation. If such lesions, which include pancreatic intraepithelial neoplasms, mucinous cystic neoplasms, IPMNs, and others, are surgically removed before they acquire the ability to invade, development of cancer can be impeded [29,129]. Several factors that differentiate PC from other pancreatic conditions and from healthy individuals, such as serum glycolipids and proteins, inflammatory and growth factors, autoantibodies, cytokines and chemokines, adhesion molecules, metabolites and DNA/RNA-based alterations, have been proposed as novel biomarkers for the early diagnosis of PC [11,13,130]. However, these have not yet been implemented in clinical practice, since evidence of their clinical value from large-scale studies is still missing [131]. The only routinely used biomarker for PC at present is serum CA 19–9, which is a marker for the confirmation of PC diagnosis and disease monitoring rather than for early screening. CA 19–9 bears the problems, that it is not expressed in individuals who belong to the Lewis blood group Le(a−b−) (8–10% of the Caucasian population), and has suboptimal sensitivities and specificities for detecting PC (79–81% and 82–90%, respectively) [132,133].

In a current review, Singhi and Wood postulate that survival in PC will improve most profoundly through the diagnosis of high-risk lesions *before* their advancement to cancer. This could be achieved by a combination of several types of biomarkers to reliably distinguish high-risk from low-risk pre-cancerous lesions, and, moreover, by finding ideal biospecimens that represent possible multifocal and genetically heterogeneous precancerous lesions [29]. Recent research efforts in PC diagnostics have focused on serum biomarkers, with special attention on CTCs, cfDNA and EVs (reviewed by [11]). It is, however, worth considering stool biomarkers as a reasonable alternative. Stool sampling is straightforward, non-invasive, can be done at home, and has the possibility of combining several types of biomarkers [134]. Since cancers of the gastrointestinal tract are in direct contact with the intestinal lumen, tumor cells as well as cfDNA and EVs are shed into the fecal mass and can be analyzed from stool. Although PC is not in direct contact with the intestinal lumen, its tumor cells, cfDNA and EVs are shed into pancreatic juice, which enters the duodenum via the pancreatic ducts. Therefore, DNA- and RNA-based biomarkers for PC could be analyzed from stool instead of conducting invasive procedures like pancreatic juice sampling or tissue biopsy. Compared to serum, stool has the advantage that besides other markers, also gut microbiota markers can be analyzed. On the other hand, stool DNA analysis bears the problem of digestive enzymes present in the intestinal tract, which can break down nucleic acids during gut transit [135]. This degradation of DNA can be impeded or attenuated by using DNA stabilizing reagents that inactivate DNases. It is therefore necessary to meticulously choose the methods of sampling and storing of stool, as well as DNA extraction, all of which have an impact on the results of stool DNA analysis. This is especially true in the case of gut fecal microbiome studies, as recently reviewed by Wu et al. [136]. In colorectal cancer, stool DNA screening tests are already in use [137], and stool microbiota markers are under evaluation [138]. In PC, no early screening methods are available to the general population, and research efforts for the utilization of stool testing with DNA/RNA-based and/or microbiota-based biomarkers are relatively scarce. However, promising results of recent studies have indicated the feasibility of fecal microbiota-based screening [119,139]. Thus, the analysis of stool bears great potential of establishing novel biomarkers for early screening and diagnosis, for prognosis and disease monitoring of PC.

## 5. Conclusions

The oncogenomics of PC tissue consist of both complex cytogenetics and mutations in numerous driver genes, of which *KRAS*, *CDKN2A*, *TP53* and *SMAD4* are the most prevalent. Likewise, epigenetic changes and alterations in ncDNA/ncRNA contribute to cancer development. These have an impact on tumor suppressors and oncogenes, important epigenetic regulators, developmental genes, and major signaling pathway genes of the pancreas, as well as those involved in the hallmarks of cancer. The fact, that these oncogenomic changes can be detected in tumor-derived cells, cfDNA/RNA or EVs present in different body fluids, enables their use as biomarkers for non-invasive early screening. For this, stool sampling could be a choice in the near future, having the advantage of being simple, cost-effective, and convenient. Stool tests are already in use for CRC, but in PC, there is still a way to go. Early efforts in testing the suitability of driver mutations as stool biomarkers readily detected mutant *KRAS* and *TP53* in stools of patients with PC, but also in stools of patients with BPD. Further studies examining the same markers show higher diagnostic value, but cannot outperform CA-19–9. Combining *KRAS* and *TP53* mutation detection with other markers is therefore suggested to increase PC detection rates. With epigenetic markers, improved but still insufficient outcomes have been achieved by combined screening of stool DNA for mutant *KRAS* and methylated *BMP3*. In addition, aberrantly expressed miRNAs in stool can be used as markers for the detection of PC, and for the differentiation between PC and other pathologic conditions of the pancreas. Comparatively good results have been achieved by the combination of several miRNA markers, such as miR-181b and -210, and miR-21, -155 and -216. A novel approach to cancer screening is the use of gut microbiota as biomarkers, based on their altered abundancies, composition, and diversity in cancer. Possible associations between genomic and microbiota alterations could be exploited for the identification of novel cancer biomarkers. For such efforts, stool represents an ideal source for the simultaneous screening of both oncogenomic and microbiota markers. At present, stool screening for the early diagnosis of PC is still in its initial stage and needs improvement. Special attention needs be paid on developing reliable markers for high-grade precancerous lesions. Analytical methods need to be improved, sensitivities and specificities of the markers need to be increased, and better marker panels need to be developed. For this, further investigations and evaluations through large-scale studies should be undertaken.

## Figures and Tables

**Table 1:** Overview of cytogenetic alterations and commonly altered oncogenic driver genes in pancreatic cancer. Compiled from [22,23,24,25,26,28].

	Numerical Aberrations and Commonly Altered Oncogenic Driver Genes in PDAC ^1^				
Chr. nr.	Gains [22,23,24,25,26]	Common Amplified Oncogenic Drivers [26,28]	Losses [22,23,24,25,26]	Common Deleted Oncogenic Drivers [26,28]	Common Break Points [23]
**1**	1q+, **amp(1q)** (33%)		1p−	*ARID1A*, 1p36.11 (6%)	1q, 1p32, 1q10
**2**	+2				
**3**	3q+		3p−	*PBRM1*, 3p21.1 (4%)	
**4**			**−4** (25–38%), 4p-, 4q−		
**5**	5p+				
**6**	6p		**−6** (25–38%), 6−, **−6p** (41%), **−6q** (51%)		6p21
**7**	**+7** (25–38%), 7p+, 7q+	*BRAF*, 7q34 (3%)		*MLL3*, 7q36.1 (4%)	7p22
**8**	8p+, 8q+	*FGFR1*, 8p11.23 (5%), *MYC*, 8q24.2 (5%)	8p−, **−8p** (28%)		8q10
**9**			**−9** (25–38%), 9p−, 9−, **−9p** (48%)	***CDKN2A***, 9p21.3 (30%)	
**10**				*PTEN*, 10q23.31	
**11**	+11, 11q+		11q−	*ATM*, 11q22.3 (5%)	
**12**	12p+, 12q+	***KRAS***, 12p12.1 (93%), *MDM2*, 12q15 (2%)	**−12** (25–38%)		
**13**			−13, 13q−	*BRCA2*, 13q13.1 (4%)	13q10
**14**					14q10
**15**	15q+		15q−		
**16**				*PALB2*, 16p12.2 (1%)	
**17**	17q+	*ERBB2*, 17q12	**−17** (25–38%), 17p−, 17−, **−17p** (64%), **−17q** (31%)	***TP53***, 17p13.1 (72%), *BRCA1*, 17q21.31 (1%)	17p11, 17q10
**18**	18q+	*GATA6*, 18q11.2 (9%)	**−18** (60%), 18q−, 18−, **−18p** (32%), **−18q** (71%), CEP18−	***SMAD4***, 18q21.1 (32%)	
**19**	19q+	*AKT2*, 19q13 (6%)	19p−	*MLL4*, 19q13.12 (4%)	19q13
**20**	**+20** (25–38%), 20q+	*GNAS*, 20q13 (8%)	20p−		
**21**			**−21** (25–38%), 21q−, 21−		
**22**			**−22** (25–38%), 22−		
**X**			**−X** (25–38%)		
**Y**			**−Y** (25–38%)		
**Structural aberrations in PDAC** [27]					
**Interchromosomal**				**14.8%**	
**Intrachromosomal**				**85.2%**	
Inversions				14.7%	
Deletions				12.5%	
Fold-back inversions				5.2%	
Amplified inversions				3.1%	
Tandem duplications				1.6%	
Duplications				1.1%	
Other				52.8%	

“+” = gain of, “−“ = loss of a chromosome, part of a chromosome or a chromosome arm; Chr. nr. = Chromosome number, CEP18 = pericentromeric region of chromosome 18, PDAC= pancreatic ductal adenocarcinoma, ^1^ references in square brackets, frequencies in round brackets, frequent aberrations and major driver genes in bold.

**Table 2:** Overview of differentially methylated genes in PDAC tissue. Compiled and modified from [41,52] and references therein.

Gene Nature	Meth. Status ^1^	Gene Names and Categories		
		**Cancer Genes** [52] **^2^**		
Tumor suppressor	+	*UCHL1* (100%), *MDF-1* * (96%), *SPARC/ON* (94%), *PENK* * (93%), *RPRM* (91%), *mi-R9-1* * (89%), *ADAMTS* ** (88–90%), *CCND* (86%), *SIP1* (73%), *BNC1* (65–78%), *PCDH10* (61%), *SOCS-1* (57%), *APC*, *RAR-β* (56%), *CDKN2A* (33%), *ATP4A*, *BMP3*, *BRCA1*, *CADM1*, *Cyclin D2*, *DNAJC15*, *FOSB*, *GSG1*, *KLF6*, *KLF10*, *miR-506*, *MLH1*, *PARP6*, *RASSF1A*, *ZPF82*, *ZNF415*		
	−	*SERPINB5* (87%), *CLDN4* (85%), *LCN2* * (85%), *SFN* (85%), *MUC4* ^2^ (80%), *TFF2* (65%), *PSCA* ** (30%), *MAP4K4* **, *SULT1E1*		
	+/−	*CDKN1C* (78%), *FOXE1* (64%)		
Oncogene	+	*mi-R9-1* * (89%), *ADAMTS* ** (87.5–90%), *KRAS* (33%)		
	−	MUC4^**^ (80%), *S100P* *** (57%), *S100A4* (50%), *PSCA* ** (30%), *IGF2BP3*, *MAP4K4* **, *MSLN*		
		Epigenetic regulatory genes [41]		
		Writers	Readers	Erasers/editors
DNA methylation	+	*DNMT3A*	*MBD1*, *ZMYM4*	*IDH2*, *MGMT*
	−	*DNMT1*	*CHD2*	*APOBEC1*, *TET3*
	+/−	*DNMT3B*	*CHD7*, *ZBTB38*, *ZMYM6*	
Histone methylation	+	*EHMT2*, *KMT2D*, *MECOM*, *PRDM8*, *PRDM12*, *PRDM13*, *PRDM14*, *SETBP1*, *SETD7*, *SETMAR*, *SMYD2*, *WHSC1L1*	*DNMT3A*	*KDM3A*, *KDM6B*
	−	*EZH2*, *PRDM2*, *PRDM11*, *PRDM15*, *WHSC1*	*ATXN7*, *CHD2*, *DHX30*, *EHMT2*, *GATAD2A*, *ZMYM8*	*KDM2A*, *KDM2B*, *KDM3B*, *KDM4B*
	+/−	*EHMT1*, *KMT2C*, *PRDM1*, *PRDM4*, *PRDM6*, *PRDM7*, *PRDM16*, *SETD3*, *SMYD3*	*CBX5*, *CHD7*, *EHMT1*, *UHRF1*	
Histone acetylation	+	*KAT2A*	*BRD4*	*HDAC11*
	−	*GTF3C1*, *NCOA2*, *NCOA7*	*ATXN7*, *BRD1*, *BRD3*, *DHX30*, *GATAD2A*, *ZMYM8*	*HDAC5*, *HDAC9*, *SIRT6*, *SIRT7*
	+/−	*CREBBP*, *KAT6B*, *NCOA1*		*HDAC4*
Arginine methylation	+	*PRMT6*		
	+/−	*PRMT8*		
Chromatin remodeler	+	*SMARCA2*		
	−	*CHD2*, *DPF3*, *SMARCD3*, *TTF2*		
	+/−	*ARID1B*, *CHD7*, *CHD8*		
Histone protein	+	*HIST3H2BB*, *HIST2H2BF*, *HIST1H2BI*, *HIST1H3B*, *HIST1H3E*, *HIST1H3F*, *HIST1H3G*, *HIST1H4F*		
	−	*H1F0*, *H1FOO*, *HIST1H1E*, *HIST1H2AG*, *HIST1H2APS1*, *HIST1H2BA*, *HIST1H2BC*, *HIST1H2BN*, *HIST1H3C*, *HIST1H3H*, *HIST1H4H*		
	+/−	*HIST3H2A*		
		Developmental and signaling genes [41]		
Homeobox-containing genes	+	*HHEX*, *HOPX*, *HOXA*, *HOXC HOXD*, *IRX2*, *IRX4* (68%), *MSX*, *PPRX*, *SHOX2*, *SOX15*, *ZEB1*		
	−	*TGIF1*, *TGIF2*, *ZEB1*		
	+/−	*HOXB*, *PAX*		
Pancreatic development	+	*FOXA1*, *GATA3*, *HLX*, *ISL1*, *MEIS2*, *NEUROG3*, *NKX2-2*, *NKX6- 1*, *PAX6*		
	−	*HNF4A*		
	+/−	*HNF1B*, *MMP2*, *MMP9*, *MNX1*, *NKX6-2*, *ONECUT1*, *SOX9*		
Pancreatic signaling	+	*EGF*, *FGF10*		
	−	*HGFAC*		
	+/−	*NOTCH1*		
		Other differentially methylated genes [41]		
Genes associated with patient survival patient survival	n.a.	*ATP4A*, *FOSB*, *GSG1*, *KLF6*		

^1^ Meth. = methylation status, + = hypermethylated, − = hypomethylated, n.a. = not available, ^2^ methylation frequencies in brackets, where available (source: [52]), * partial tumor suppressor, ** tumor suppressor or oncogene, dependend on tissue or cancer type, *** oncogene, might also have tumor suppressor function.

**Table 3:** Major differentially expressed miRNAs, lncRNAs and circRNAs in PC tissue and cell lines, their targets and biological functions. Compiled and modified from [55,57,58,59] and references therein, with additions from [60,61,62,63,64,65,66,67,68,69].

Upregulated miRNAs (Oncogenic Function)	Target Genes/Pathways	Biological Functions in PC
miR-10a	*HOXA1*	🠙 invasion
miR-15b	*SMURF2*	🠙 EMT
miR-17-5p	*E2F4*, RBL2/E2F4-complex	🠙 proliferation
miR-21	*Bcl-2*, *FasL*, *Fox01*, *PDCD4*, *PTEN*, *RECK*, *TPM1*, *TIMP3*	🠙 chemoresistance, 🠙 invasion, 🠙 proliferation, 🠙 metastasis, 🠙 EMT, 🠛 apoptosis
miR-23a	*APAF1*, *FZD5*, *HNF1B*, *TMEM92*	🠙 proliferation, 🠛 apoptosis
miR-23b	*JAK2*, *PI3K*, *PTEN*, *ATG12*, *AKT*/NF-κB	🠙 proliferation, 🠙 tumor growth, 🠙 migration, 🠙 invasion
miR-24	*Bim*, *FZD5*, *HNF1B*, *TMEM92*	🠙 cell growth, 🠙 EMT
miR-27a	*Sprouty2*	🠙 proliferation, 🠙 migration, 🠙 colony formation
miR-29a	Wnt/β-catenin	regulates transcription factors
miR-92a	*DUSP10*	🠙 proliferation
miR-155	*Foxo3a*, *KRAS*, *ROS*, *SEL1L*, *MLH1*, *SOCS1*, *TP53INP1*	🠙 invasion, 🠙 migration, 🠙 proliferation, 🠙 tumor growth
miR-181a	*PTEN*, *MAP2K4*, *TNFAIP1*	🠙 migration, 🠙 proliferation
miR-181b	*BCL-2*, *CYLD*	🠙 chemoresistance
miR-196a	*NFKBIA*, *ING5*	🠙 migration, 🠙 proliferation
miR-191	*USP10*	🠙 proliferation
miR-210	Pancreatic stellate cells	🠙 EMT, 🠙 invasion
miR-214	*ING4*	🠛 chemosensitivity
miR-221	*p27kip1*, *PTEN*, *p57kip2*, *PUMA*, *TIMP2*, *TRPS1*	🠙 invasion
miR-222	*p57*, *MMP2*, *MMP9*	🠙 invasion
miR-223	*FBw7*	🠙 EMT
miR-320a	*PDCD4*	🠙 EMT, 🠛 apoptosis
miR-365	*BAX*, *SHC1*	🠛 apoptosis
**Downregulated miRNAs** **(tumor suppressive function)**	**Target Genes/Pathways**	**Biological Function in PC**
miR-26a	*CCNE2*, *TP53*	🠛 proliferation, 🠛 phosphorylation of *TP53*
miR-29c	*ITGB1*, *MMP2*, *FRAT2*, *LRP6*, *FZD4*, *FZD5*	🠛 cell growth, 🠛 migration, 🠛 invasion, 🠛 metastasis
miR-30a	*FOXD1*	🠛 proliferation, 🠛 cell cycle, 🠙 apoptosis, 🠙 chemosensitivity
miR-31	*BCL2*	🠛 chemoresistance
miR-33a	*AKT*, β-catenin, PIM-kinase	🠛 proliferation
miR-34a	*BCL2*, *NOTCH1*, *NOTCH2*	🠛 proliferation, 🠛 invasion, 🠙 apoptosis
miR-96	*NUAK1*	🠛 proliferation, 🠛 invasion, 🠛 migration
miR-100	*FGFR3*	🠙 chemosensitivity, 🠛 proliferation
miR-101-3p	*RRM1*	interferes with DNA synthesis
miR-107	*CDK6*	🠛 proliferation
miR-130b	*STAT3*	🠛 proliferation, 🠛 invasion
miR-141	*MAP4K4*, *TM4SF1*, *YAP1*	🠛 proliferation, 🠛 colony formation, 🠛 migration, 🠛 invasion
miR-143	*ARHGEF1*, *ARHGEF2*, *KRAS*	🠛 invasion, 🠛 migration, 🠛 metastasis
miR-145	*KRAS*, *RREB1*, *ROR*, *MUC13*	🠛 cell cycle, 🠛 proliferation, 🠛 invasion
miR-146a	*EGFR*, *IRAK1*, *MTA-2*	🠛 invasion
miR-148a	*DNMT1*, *CCKBR*, *BCL-2*, *CDC25B*	🠛 proliferation, 🠛 metastasis, 🠛 cell growth
miR-148b	*AMPKα1*, *DNMT1*	🠛 cell growth, arrests cell cycle, modifies methylation of tumor suppressors
miR-150	*MYB*, *MUC4*	🠛 proliferation, 🠛 invasion, 🠛 migration, 🠙 intercellular adhesion, 🠙 apoptosis
miR-200c	*MUC4*, *MUC6*, E-cadherin	🠛 invasion, 🠙 proliferation
miR-211	*RRM2*	🠛 invasion
miR-216a	*JAK2*, *Beclin-1*	🠛 proliferation, 🠙 apoptosis, 🠙 radiosensitivity
miR-217	*KRAS*, *SIRT1*	🠛 cell growth, 🠛 colony formation, regulation of EMT
miR-335	*OCT4*	🠛 tumor development, 🠛 clonogenic expansion
miR-365	*BAX*, *SHC1*	🠙 chemorsistance
miR-375	*PDK1*	🠛 cell growth, 🠛 proliferation, 🠙 apoptosis
let-7	*ZEB1/*N-cadherin	🠛 EMT, 🠛 invasion
**Upregulated lncRNAs** **(oncogenic function)**	**Target Genes/miRNAs/Pathways**	**Biological Function in PC**
H19	*HMGA2*, *E2F*, let-7, miR-675, -194	🠙 proliferation, 🠙 tumor growth, 🠙 metastasis
HOTAIR	*EZH2*, miR-34a	🠙 proliferation, 🠙 chemoresistance
HOTTIP	HOXA9, HOXA13, miR-137	🠙 cell growth, 🠙 invasion, 🠙 chemoresistance, modulates stem cells
MALAT-1	*Sox-2*, *EZH2*, miR-200c, -216a, -217, Hippo-YAP	🠙 cell growth, 🠙 migration, 🠙 invasion, 🠙 metastasis
PVT1	*p21*, miR-20a-5p, -448, -519, HIF-1, YKT6, RAB7, VAMP3, ULK1	🠙 proliferation, 🠙 migration, 🠙 chemoresistance
**Downregulated lncRNAs** **(tumor suppressive function)**	**Target Genes/miRNAs/Pathways**	**Biological Function in PC**
GAS5	*CDK6*, miR-32, -181c, -221/SOCS3	🠛 metastasis, 🠙 chemosensitivity, reverses EMT
MEG	c-Met, PI3K/AKT	🠛 proliferation, 🠛 migration, 🠛 invasion
**Upregulated circRNAs** **(oncogenic function)**	**Target genes/miRNAs/pathways**	**Biological function in PC**
ci-RS-7	EGFR, STAT3; Sponges miR-7.	🠙 proliferation, 🠙 invasion
circEIF6	SLC7A11, PI3K/AKT; Sponges miR-557.	🠙 proliferation, 🠛 apoptosis
circFOXK2	ANK1, GDNF, PAX6, NUF2, PDXK; Sponges miR-942.	🠙 proliferation, 🠙 migration, 🠙 invasion
circBFAR	MET/PI3K/Akt.; Sponges miR-34b-5p.	🠙 proliferation, 🠙 motility
circ-ASHL2	Notch 1; Sponges miR-34a.	🠙 proliferation, 🠙 invasion, 🠙 angiogenesis
circHOT1	E2F3; Sponges miR-125a, -330, -26b and -382.	🠙 proliferation, 🠙 migration, 🠙 invasion
circRNA_100782	IL6, STAT3; Sponges miR-124	🠙 proliferation, 🠙 tumor growth
hsa_circ_0071036	Bcl-2, caspase-3; Sponges miR-489	🠙 proliferation, 🠙 invasion, 🠙 tumor growth
hsa_circ_0007534	Sponges miR-625 and -892b.	🠛 apoptosis
**Downregulated** **circRNAs** **(tumor suppressive function)**	**Target Genes/miRNAs/Pathways**	**Biological Function in PC**
circNFIB1	PIK3R1, VEGF-C; Sponges miR-486-5p	🠛 lymph node metastasis
hsa_circ_001587	SLC4A4, MMP-2, MMP-9, MCM2, VEGF; Sponges miR-223.	🠛 proliferation, 🠛 migration, 🠛 invasion, 🠛 angiogenesis

🠙 = increase, 🠛 = decrease or inhibition, EMT = epithelial to mesenchymal transition.

**Table 4:** Detection of pancreatic cancer mutations, methylations and miRNA changes in stool.

Reference	Study Population	Controls	Methods	DNA-/RNA-Based Markers	Detection Rate in Stool/ST and SF	Detection Rate in TT/PT/PJ	Other Markers	Main Findings/Authors’ Conclusions
Caldas et al., 1994 [95]	11 PDAC, 3 CCA, 3 CP, 1 PTu	n.a.	Plaque hybr. assay	*KRAS* codon 12 in stool and tissue	In 55% of PDAC, in 67% of CCA, in 33% of CP.	TT/PT: in 100% of PDAC, 67% of CCA, 65% of duct lesions.	n.a.	*KRAS* mutations from PC cells and from abnormal duct epithelium can be detected in stool; potential use for screening of PDAC and precursor lesions.
Berndt et al., 1998 [96]	42 PDAC, 1 CAC, 1 CA, 7 PAC, 1 NEC, 2 PI, 7 CP	6 HC	Mut.-enr. PCR and rev. dot-plot hybr. in microplates	*KRAS* in stool and tissue	In 40% of PDAC, in 100% of CAC, in 33% of CP; ST 42.3%, SP 66.7%.	TT/PT: in 91% of PDAC, 71% of PAC, 67% of CP.	Serum CA 19–9 and CEA	Diagnostic ST of *KRAS* in stool is only 40%, which is similar to CEA but much lower than CA 19–9. Establishment of marker combinations for stool testing is necessary.
Wenger et al., 1999 [97]	36 PDAC, 7 PAC, 1 CAC, 2 PI, 5 CP	10 HC	Allele-specific capture probes, mut.-enr. PCR	*KRAS* in stool and tissue	In 20% of PDAC, in 100% of CAC, in 40% of CP.	TT/PT: in 78% of PDAC, 100% of CAC, 14% of PAC, 20% of CP.	Serum CA 19–9 and CEA	Mut. *KRAS* analysis in tissue did not distinguish between benign and malignant pancreatic disease. Only 20–40% of PC cases can be traced back from stool samples. Stool analysis could still be useful to detect more cases and increase survival.
Pezzilli et al., 2006 [98]	PDAC, CAC, PET, CP, pseudocysts, benign congenital pancreatic mass	n.a.	PCR amplification	*KRAS* codon 12 in stool and blood	No detection	No detection	n.a.	*KRAS* mutation analysis in blood and stool is not useful for differentiating benign and malignant pancreatic masses. Further studies are needed to find simple and useful genetic markers for the detection of pancreatic malignancy.
Lu et al., 2002 [99]	201 PC or BPD	60 HC	PCR-RFLP, PCR-SSCP	*KRAS* and *TP53* in stool and PJ	Mut. *KRAS* in 88% of PC, 51.1% of BPD, 19.6% of HC; mut. *TP53* in 37.1% of PC and 19.1% of CP.	PJ: mut. *KRAS* in 87.8% and 23.5%, mut. *TP53* in 47.4% and 12.5% of PC and BPD.	n.a.	*KRAS* mutation analysis in pancreatic juice might be used in PC diagnosis. Combined *KRAS* and *TP53* mutation analysis in stool can improve PC screening.
Wu et al., 2006 [100]	31 PC for fecal analysis, 48 PC for serum analysis	85 controls with benign digestive disorders	PCR-RFLP, PCR-SSCP	*KRAS* and *TP53* in stool	Mut. *KRAS* in 77.4% of PC and 18.2% of controls; mutated TP53 in 25.8% of PC and 4.71% of controls.	n.a.	Serum CA 19–9, CA 242, CA 50, CEA	Fecal *KRAS* and *TP53* mutations do not differ between tumor subgroups, which indicates an early role in tumorigenesis. The diagnostic value of CA 19–9 and CA 242 could be improved by combination with fecal *KRAS* analysis.
Hwang et al., 2011 [101]	14 PC, 6 IPMN	20 HC	Hybrid capture enrichment of *KRAS*; QuARTS	*KRAS* in stool	62% ST for PC and 83% for IPMN (at 90% SP cutoff).	n.a.	n.a.	Pancreatic neoplasia can be detected by stool screening, but further studies using genetic and epigenetic alterations complementary to *KRAS* are needed.
Wang et al., 2018 [102]	88 PC, 35 CP, 19 BPD	3 HC	Magnetic nanoparticle trace capture probe and PCR	*KRAS* in stool and tumor tissue	Mut. *KRAS* in 81.8% of PC and 18.5% of BPD, 0% of HC; ST and SF for detecting PC: 81.8% and 81.5%.	n.a.	Serum CA 19–9	ST and SF of fecal mut. *KRAS* for detection of PC was slightly higher than that of serum CA 19–9. By combining both markers, sensitivity could be increased to 97.9% while specificity stayed the same.
Haug et al., 2007 [103]	875 unselected older adults	n.a.	Mut.-enr. PCR and allele-specific hybr. reaction	*KRAS* codons 12 and 13 in stool	8% overall prevalence of mut. *KRAS.*	n.a.	n.a.	Tentative association between decreased fecal pancreatic elastase 1 and mut. *KRAS* in stool, but no *KRAS* mutations detected in cases that later developed CRC. This assay could be used for early detection of PC, but not for CRC screening.
Kisiel et al., 2012 [92]	58 PDAC	65 HC	Sequence specific gene capture (stool), MSP (tissue and stool); QuARTS	Mut. *KRAS* and meth. *BMP3*, *NDRG4*, *EYA4*, *UCHL1*, *MDFI*, *Vimentin*, *CNTNAP2*, *SFRP2*, *TFPI2* in stool and tissue	Meth. *BMP3* detected 51%, mut. *KRAS* detected 50% and combination of both detected 67% of PDAC.	n.a.	n.a.	PC can be detected from stool assay of methylated gene markers; *BPM3* performed well alone; combining it with mut. *KRAS* increased detection rate for PDAC.
Link et al., 2012 [106]	15 PC, 15 CP	15 HC	Taq-Man miRNA assay	miR-21, -143, -155, -196a, -210, -216a, -375	Lower expression of miR-216a, -196a, -143 and -155 in PC compared to HC.	n.a.	n.a.	Differentially expressed miRNAs can be detected in stool of PC patients. This may be used as biomarker for PC screening.
Ren et al., 2012 [107]	29 PC, 22 CP	13 HC	Taq-Man miRNA assay	miR-16, -21, -155, -181a, -181b, -196a and -210	mi-RNAs discriminated PC from HC; miR-181b at a ST and SF of 84.6% and 51.7%; miR-210 at a ST and SF of 84.6% and 65.5%.	n.a.	n.a.	Fecal miRNAs may be used as novel biomarkers for PC screening.
Yang et al., 2014 [108]	30 PDAC, 10 CP	15 HC	miRNA expr. analysis with qRT-PCR	miR-21, -155, -196a, -216 and -217	Sign. higher expr. of miR-21 and -155 and lower expr. of miR-216 in PC compared to HC; ST of miR-21 and -155: 93.33%; ST and SF of miR-21, -155 and -216: 83.33% and 83.33%.	TT/PT/PJ: sign. higher expr. of miR-21 and -155; sign. lower expr. of miR-216 in PDAC compared to CP.	n.a.	MiRNA stool sampling and analysis is highly reproducible. Consistency in expression levels of miR-21, -155 and -216 in matched PC tissue, PJ, and stool samples. Combination of two or three miRNA markers yields enough ST and SF for their possible use as biomarkers for PC screening.

BPD = benign pancreatic disease, CA = cystadenoma, CAC = cystadenocarcinoma, CP = chronic pancreatitis, CRC = colorectal cancer, expr. = expression, HC = healthy controls, hybr. = hybridization, MSP = real-time methylation-specific PCR, meth. = methylated, mut. = mutant, mut.-enr. = mutant-enriched, NEC = neuroendocrine carcinoma, n.a. not applicable, PA = papillary adenocarcinoma, PAC = periampullary carcinoma, PC = pancreatic cancer, PDAC = pancreatic ductal adenocarcinoma, PET = pancreatic endocrine tumor, PI = pancreatic insulinoma, PJ = pancreatic juice, PT = pancreatic tissue, PTu = pancreatic tuberculosis, QuARTS = quantitative allele-specific real-time target and signal amplification, qRT-PCR = quantitative Reverse Transcription PCR, RFLP = restriction fragment length polymorphisms, SF = specificity, sign. = significantly, SSCP = single-strand conformation polymorphism, ST = sensitivity, TT = tumor tissue.

## Data Availability

Not applicable.
